# Visual Improvement and Optical Quality Changes After Keratoplasty in Macular Corneal Dystrophy in Romania

**DOI:** 10.22336/rjo.2026.07

**Published:** 2026

**Authors:** Ana Maria Arghirescu, Alina Gabriela Gheorghe, Maria Cristina Marinescu, Liliana Mary Voinea, Radu Ciuluvică

**Affiliations:** 1 Department of Ophthalmology, “Prof. Dr. Mircea Olteanu” Clinical Institute of Ophthalmological Emergencies, Bucharest, Romania; 2 Doctoral School, “Carol Davila” University of Medicine and Pharmacy, Bucharest, Romania; 3 Department of Ophthalmology, “Carol Davila” University of Medicine and Pharmacy, Bucharest, Romania; 4 Medical Physiology Discipline, “Carol Davila” University of Medicine and Pharmacy, Bucharest, Romania; 5 Department of Anatomy, Faculty of Dental Medicine, “Carol Davila” University of Medicine and Pharmacy, Bucharest, Romania

**Keywords:** macular corneal dystrophy, keratoplasty, anterior segment optical coherence tomography, higher-order aberrations, Strehl ratio, AS-OCT = anterior segment optical coherence tomography, BCVA = best corrected visual acuity, CYL = cylinder, DALK = deep lamellar anterior keratoplasty, HOA = high order aberrations, MCD = macular corneal dystrophy, MPP = mean pupillary power, PK = penetrating keratoplasty, PSF = point spread functions

## Abstract

**Objective:**

To evaluate visual and optical outcomes after penetrating keratoplasty in eyes with macular corneal dystrophy in Romania.

**Methods:**

This observational study included 11 eyes from 6 patients with macular corneal dystrophy who underwent penetrating keratoplasty. Preoperative and 6-month postoperative assessments included best-corrected visual acuity, central corneal thickness, and anterior segment optical coherence tomography parameters obtained with the CSO MS-39, including higher-order aberrations at 3 mm, mean pupil power, cylinder, and point spread function expressed as the Strehl ratio. Continuous variables were summarized as mean ± standard deviation. Preoperative and postoperative values were compared using the Wilcoxon signed-rank test. Associations between preoperative central corneal thickness and other baseline variables were assessed using Spearman correlation.

**Results:**

Mean best-corrected visual acuity improved from 0.123 ± 0.090 preoperatively to 0.755 ± 0.082 at 6 months (p = 0.0010), corresponding approximately to an improvement from 0.91 to 0.12 logMAR. High-order aberrations at 3 mm increased from 0.269 ± 0.106 to 0.777 ± 0.523 (p = 0.0010), and cylinder magnitude increased from -1.42 ± 0.44 diopters to -8.15 ± 4.10 diopters (p = 0.0010). The Strehl ratio decreased from 0.214 ± 0.039 to 0.080 ± 0.024 (p = 0.0010). Mean pupil power increased from 42.60 ± 0.88 to 44.58 ± 2.55 without statistical significance (p = 0.0537). Preoperative central corneal thickness was positively correlated with age (rho = 0.699, p = 0.0167).

**Discussion:**

Our study underscores that even if visual quality metrics, such as higher-order aberrations and the point spread function, have decreased after keratoplasty, alongside a significant increase in astigmatism, penetrating keratoplasty still plays a very important role in restoring visual independence in patients with advanced macular dystrophy.

**Conclusions:**

Penetrating keratoplasty in macular corneal dystrophy was associated with substantial improvement in visual acuity at 6 months, despite worsening in several optical quality metrics derived from anterior segment optical coherence tomography. These findings suggest that functional visual gain after keratoplasty may occur even when postoperative optical regularity does not improve.

## Introduction

Macular corneal dystrophy (MCD) is a rare, bilateral, progressive stromal corneal dystrophy characterized by abnormal deposition of poorly sulfated glycosaminoglycans within the corneal stroma that can also involve the Descemet membrane and the endothelium, leading to progressive corneal clouding and visual impairment. With the progressive nature of corneal stromal dystrophies, the visual acuity and visual quality of otherwise healthy patients gradually decline, with clinical onset in the first or second decade of life [1]. In a major review of MCD, the authors noted a mean age of first penetrating keratoplasty of 41 ± 4 years [2,3]. The treatment course of macular corneal dystrophy (MCD) includes penetrating keratoplasty (PK) or deep anterior lamellar keratoplasty (DALK).

Regarding prevalence, macular corneal dystrophy (MCD) appears to be very rare in Europe. The available PubMed data suggest that MCD is rare across Europe but unusually frequent in Iceland, likely reflecting founder effects and population structure [4].

The long-term visual outcomes post-keratoplasty in these patients vary with several important factors: the type of dystrophy (with different stromal dystrophies associated with different rates of graft recurrence) and the type of corneal transplantation (DALK or PK). A 2025 meta-analysis by Awad et al. revealed that PK may achieve better early postoperative vision in some series, likely because there is no host-donor stromal interface. But by about 1 year, after suture removal, many studies report comparable final BCVA between DALK and PK [5,6]. It is worth noting, however, that even if DALK is usually preferred postoperatively because it provides similar long-term visual outcomes with lower rejection risk and better endothelial survival, PK remains important when Descemet membrane or endothelium is compromised, when deep scarring precludes successful lamellar dissection, or when the surgeon judges that full-thickness transplantation will give the best anatomic result, which was our case.

## Methods

This retrospective observational study was conducted at “Prof. Dr. Mircea Olteanu” Clinical Institute of Ophthalmological Emergencies (tertiary referral center). The study included 11 eyes from 6 patients diagnosed with macular stromal dystrophy, without any prior ocular surgery or known ocular diseases, who underwent penetrating keratoplasty between January 2022 and March 2025 at “Prof. Dr. Mircea Olteanu” Clinical Institute of Ophthalmological Emergencies.

The study was done in accordance with the principles of the Helsinki Declaration and was approved by the Ethics Committee of “Prof. Dr. Mircea Olteanu” Clinical Institute for Ophthalmological Emergencies (1028/19.03.2026). Written informed consent was obtained from all patients before the intervention.

All patients underwent complete ophthalmological examination and anterior segment optical coherence tomography preoperatively and at the 6-month mark. No late postoperative complications such as graft rejection, dystrophy recurrence, or secondary glaucoma were present in the cohort at the 6-month mark. Best corrected visual acuity, intraocular pressure, slit-lamp aspects, as well as AS-OCT (CSO MS-39) parameters such as mean pupillary power (MPP), cylinder power and axis (CYL), high order aberrations (HOA), and point spread function (PSF) were recorded.

### Statistical analysis

Continuous variables were summarized as mean ± standard deviation, and categorical variables as counts and percentages. The association between preoperative central corneal thickness and other preoperative variables was assessed using Spearman’s rank correlation coefficient, given the small sample size and the possibility of non-normal distribution of the studied parameters. Comparisons between preoperative and 6-month postoperative measurements were performed using the Wilcoxon signed-rank test. A p-value of < 0.05 was considered statistically significant.

## Results

A total of 11 eyes from 6 patients with macular corneal dystrophy were included in this observational study. Mean age at baseline was 46.2 ± 11.7 years (median 42.5 years, range 36-68 years). The cohort included 3 female patients (50.0%) and 3 male patients (50.0%).

Mean preoperative best-corrected visual acuity (BCVA) was 0.123 ± 0.090, improving to 0.755 ± 0.082 at 6 months postoperatively. Mean preoperative HOA at 3 mm was 0.269 ± 0.106, increasing to 0.777 ± 0.523 postoperatively. Mean preoperative mean pupil power (MPP) was 42.60 ± 0.88, compared with 44.58 ± 2.55 at 6 months. Mean cylinder changed from -1.42 ± 0.44 D preoperatively to -8.15 ± 4.10 D postoperatively. Mean point spread function (PSF) decreased from 0.214 ± 0.039 preoperatively to 0.080 ± 0.024 at 6 months.

In paired analysis, BCVA improved significantly from 0.123 ± 0.090 preoperatively to 0.755 ± 0.082 postoperatively (p = 0.0010). HOA at 3 mm increased significantly from 0.269 ± 0.106 to 0.777 ± 0.523 (p = 0.0010). Cylinder magnitude also increased significantly from -1.42 ± 0.44 D to -8.15 ± 4.10 D (p = 0.0010). PSF showed a significant reduction from 0.214 ± 0.039 to 0.080 ± 0.024 (p = 0.0010). MPP increased from 42.60 ± 0.88 to 44.58 ± 2.55, but this difference did not reach statistical significance (p = 0.0537).

**Fig. 1:**
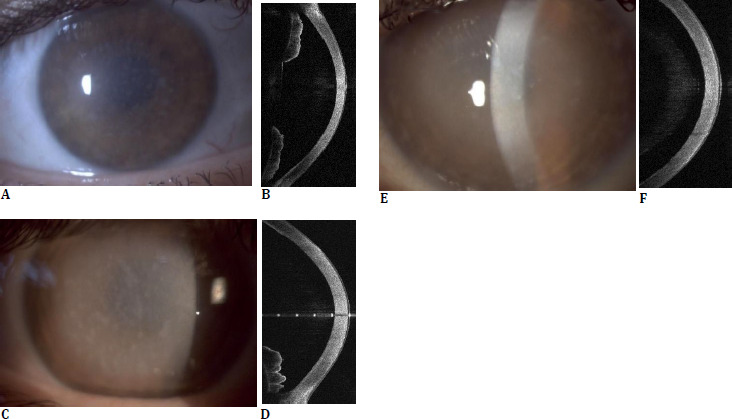
Slit lamp and AS-OCT of macular corneal dystrophy. A.P., 38-year-old woman (**A, B**); 39-year-old woman, older sister of patient A.P. (**C, D**); 46-year-old male (**E, F**)

Mean preoperative central corneal thickness (CCT) was 457.6 ± 22.5 µm (median, 454 µm; range, 429-497 µm). In Spearman correlation analysis, preoperative CCT showed a significant positive correlation with age (rho = 0.699, p = 0.0167). No significant correlations were observed between CCT and preoperative visual acuity (rho = -0.025, p = 0.9415), HOA 3 mm (rho = -0.141, p = 0.6787), MPP (rho = 0.474, p = 0.1410), or PSF (rho = 0.200, p = 0.5554). A borderline positive correlation was found between CCT and preoperative cylinder (rho = 0.591, p = 0.0556) (**Table 1, Fig. 1A-F**).

**Table 1:** Comparison between preoperative and postoperative values of best corrected visual acuity, higher-order aberrations, mean pupillary power, cylinder, and point spread function

Variable	Preoperative n	Preoperative mean ± SD	Postoperative mean ± SD	Mean change (Post − Pre)	p value
Visual acuity (BCVA)	11	0.123 ± 0.090	0.755 ± 0.082	+0.632	*0.0010*
HOA 3 mm	11	0.269 ± 0.106	0.777 ± 0.523	+0.508	*0.0010*
MPP	11	42.60 ± 0.88	44.58 ± 2.55	+1.982	0.0537
Cylinder (D)	11	-1.42 ± 0.44	-8.15 ± 4.10	-6.727	*0.0010*
PSF (Strehl ratio)	11	0.214 ± 0.039	0.080 ± 0.024	-0.134	*0.0010*

## Discussion

When expressed in logMAR terms, mean BCVA improved from 0.91 logMAR preoperatively to 0.12 logMAR at 6 months, corresponding to an average gain of approximately 0.79 logMAR. This indicates a marked improvement in visual acuity and confirms that the postoperative visual benefit was not only statistically significant but also clinically substantial.

However, it is worth noting that postoperatively, the parameters associated with visual quality suffered significant changes: the high order aberrations on the central corneal 3 mm increased (HOA 3 mm - from 0.269 ± 0.106 to 0.777 ± 0.523, p 0.001) and the point spread function measured by the Strehl ratio significantly decreased, PSF -0.214 ± 0.039 0.080 ± 0.024 0.0010). Given that a Strehl ratio closer to 1 indicates better optical quality, this finding suggests a deterioration in postoperative optical performance. In parallel, the HOA at 3 mm and the refractive cylinder increased, further supporting the presence of greater postoperative optical irregularity. The discordance between improved visual acuity and worsened Strehl ratio may indicate that the preoperative visual limitation in macular corneal dystrophy is driven predominantly by stromal opacity and light scatter. In contrast, postoperative corneal remodeling may induce aberrational changes not fully reflected in standard acuity testing.

A major limitation of our study was the small number of patients included, due to the rarity of corneal dystrophies. Macular corneal dystrophy appears to be a more frequent ophthalmological finding in the Romanian population; however, assessing its incidence and prevalence requires more data.

## Conclusions

Keratoplasty in macular corneal dystrophy was associated with a marked improvement in visual acuity at 6 months, indicating substantial functional visual benefit after surgery. However, this gain was accompanied by a deterioration in several AS-OCT-derived optical quality parameters, including higher-order aberrations, refractive cylinder, and the point spread function, as measured by the Strehl ratio. These findings suggest that postoperative visual recovery in macular corneal dystrophy may be driven primarily by restoration of corneal transparency and reduction of visually significant stromal opacity, rather than by parallel improvement in corneal optical regularity. Preoperative central corneal thickness was associated with age but showed no clear relationship with most baseline functional or optical parameters. Although limited by the small sample size, inclusion of both eyes from some patients, and short follow-up, this study supported the value of combining conventional visual acuity assessment with quantitative anterior segment optical coherence tomography metrics when evaluating outcomes after keratoplasty in macular corneal dystrophy.
